# Prenatal Organochlorine Compound Exposure, Rapid Weight Gain, and Overweight in Infancy

**DOI:** 10.1289/ehp.1002169

**Published:** 2010-10-05

**Authors:** Michelle A. Mendez, Raquel Garcia-Esteban, Mónica Guxens, Martine Vrijheid, Manolis Kogevinas, Fernando Goñi, Silvia Fochs, Jordi Sunyer

**Affiliations:** 1 Center for Research in Environmental Epidemiology, Barcelona, Spain; 2 Municipal Institute of Medical Research (IMIM-Hospital del Mar), Barcelona, Spain; 3 CIBER Epidemiologia y Salud Pública, Spain; 4 National Institute of Public Health, Athens, Greece; 5 Laboratorio de Salud Pública de Guipúzcoa, San Sebastián, Spain; 6 Universitat Pompeu Fabra, Barcelona, Spain

**Keywords:** β-hexachlorohexane, body mass index, dichlorodiphenyldichloroethylene, hexachlorobenzene, infant, obesity, polychlorinated biphenyls, prenatal, weight gain

## Abstract

**Background:**

Although it has been hypothesized that fetal exposure to endocrine-disrupting chemicals may increase obesity risk, empirical data are limited, and it is uncertain how early in life any effects may begin.

**Objectives:**

We explored whether prenatal exposure to several organochlorine compounds (OCs) is associated with rapid growth in the first 6 months of life and body mass index (BMI) later in infancy.

**Methods:**

Data come from the INMA (Infancia y Medio-Ambiente) Child and Environment birth cohort in Spain, which recruited 657 women in early pregnancy. Rapid growth during the first 6 months was defined as a change in weight-for-age *z*-scores > 0.67, and elevated BMI at 14 months, as a *z*-score ≥ the 85th percentile. Generalized linear models were used to estimate the risk of rapid growth or elevated BMI associated with 2,2-bis(*p*-chlorophenyl)-1,1-dichloroethylene (DDE), hexachlorobenzene, β-hexachlorohexane, and polychlorinated biphenyls in first-trimester maternal serum.

**Results:**

After multivariable adjustment including other OCs, DDE exposure above the first quartile was associated with doubling of the risk of rapid growth among children of normal-weight (BMI < 25 kg/m^2^), but not overweight, mothers. DDE was also associated with elevated BMI at 14 months (relative risk per unit increase in log DDE = 1.50; 95% confidence interval, 1.11–2.03). Other OCs were not associated with rapid growth or elevated BMI after adjustment.

**Conclusions:**

In this study we found prenatal DDE exposure to be associated with rapid weight gain in the first 6 months and elevated BMI later in infancy, among infants of normal-weight mothers. More research exploring the potential role of chemical exposures in early-onset obesity is needed.

The past few decades have seen dramatic increases in obesity prevalence in all age groups worldwide (Kelly et al. 2008; Wang and Lobstein 2006). There is substantial evidence that obesity risk may begin very early in life; > 40 studies have documented rapid weight gain in the first few months after birth to be associated with obesity later in life (Akaboshi et al. 2008; Blair et al. 2007; Botton et al. 2008; Chomtho et al. 2009; Demerath et al. 2009; Dubois and Girard 2006; Durmuş et al. 2010; Ekelund et al. 2006; Eriksson et al. 2008; Goodell et al. 2009; Hitze et al. 2010; Kain et al. 2009; Karaolis-Danckert et al. 2006; Lamb et al. 2010; Ong and Loos 2006; Ong et al. 2009; Taveras et al. 2009; Toschke et al. 2004) or related metabolic disorders (Cournil et al. 2009; EURODIAB Substudy 2 Study Group 2002; Harder et al. 2009; Huxley et al. 2000; Law et al. 2002; Leunissen et al. 2009). Although feeding practices likely play a role (Koletzko et al. 2009; Worobey et al. 2009), there is considerable uncertainty regarding other factors that may program this rapid infant growth.

There is emerging interest in the possibility that perinatal exposure to certain xenobiotic chemicals may be obesogenic (Grün and Blumberg 2009; Heindel and vom Saal 2009). Substantial evidence suggests that gestational tobacco exposure is related to excess weight gain and later obesity (Mendez et al. 2008; Oken et al. 2008). Laboratory studies suggest that prenatal exposure to some endocrine-disrupting compounds (EDCs) may also promote obesity. For example, despite similar dietary intakes, excess postnatal weight gain has been observed in mice exposed perinatally to bisphenol A (BPA) or organotins (Grün et al. 2006; Rubin et al. 2001; Somm et al. 2009). However, few EDCs have been examined in laboratory studies thus far. More important, there are limited empirical data linking such exposures to obesity in humans (Elobeid and Allison 2008). One recent study (Smink et al. 2008) reported an association between obesity at 6.5 years of age and prenatal hexachlorobenzene (HCB) exposure, and two reported fetal exposure to 2,2-bis(*p*-chlorophenyl)-1,1-dichloroethylene (DDE) to be associated with higher body mass index (BMI) at 3 years of age (Verhulst et al. 2009) or in adulthood (Karmaus et al. 2009). It remains uncertain whether such exposures in humans may program rapid weight gain beginning in infancy.

In this study we examined the relationship between rapid weight gain in the first 6 months of life and prenatal exposure to several organochlorine compounds (OCs) with endocrine-disrupting properties, including DDE and HCB. Because maternal weight status may modify children’s risk of obesity, perhaps due to differential feeding practices (Buyken et al. 2008) or higher OC concentrations among those with higher BMIs (Jakszyn et al. 2009), we examined possible effect modification by prepregnancy overweight/obesity (hereafter overweight) *a priori*. We also examine how rapid early weight gain and OC exposure relate to BMI status attained in the second year of life.

## Materials and Methods

Data come from the Environment and Childhood Project [Infancia y Medio-Ambiente (INMA)] in Sabadell (Catalonia, Spain), a population-based birth cohort that enrolled 657 women in 2004–2006, during the first trimester of pregnancy (Ribas-Fitó et al. 2006). Women were recruited from public health centers during their first ultrasound visit (mean ± SD, 13.5 ± 1.7 weeks’ gestation); 99.5% of Spaniards have public health insurance, and an estimated 70–90% of women use public health services during pregnancy (Regidor et al. 2008). The participation rate was 60%. Questionnaires were administered by trained interviewers during the first and third trimester, at delivery, and at 6 and 14 months after birth to assess health and feeding behaviors, health status, sociodemographic characteristics, reproductive history, and other factors. Maternal blood was collected at recruitment. Birth outcomes were available for 616 women (94%), including 599 term births (≥ 37 weeks’ gestation) eligible for this analysis. Of these, 518 (86%) had data on infant growth, OCs, and key covariates and were included in the analysis sample. Ethical approval was obtained from the Clinical Research Ethical Committee of the Municipal Institute of Health Care, and informed consent was obtained from all subjects.

### Infant anthropometry

Infant weight (nearest gram) and length (nearest 0.1 cm) were measured at birth and at 14 months by trained staff in using standard protocols (Lohman 1988). Infants below the 10th percentile of a Spanish reference were classified as small for gestational age (SGA) (Carrascosa et al. 2004). Repeated measures of infant weight throughout the first 6 months were abstracted from medical records. Weight gain from birth to 6 months was estimated in two ways. First, weight measures were used to construct sex-specific growth curves using mixed models with random intercept and slope after transformations based on fractional polynomials (Royston 1995). These curves were used to estimate weight at 6 months of age for infants not measured within ±14 days of their exact 6-month anniversary (16.2% of the sample). The correlation between measured and predicted 6-month weight was 0.96; restricting the analysis to infants with measured weights yielded very similar results (data not shown). Second, weight measured within ±1 month of the 6-month anniversary was used to estimate average daily weight gain through 6 months (95.8% of the analysis sample). Defining rapid growth as the highest tertile of average daily weight gain yielded very similar results (data not shown).

To define rapid growth, weight at birth and at 6 months of age was standardized based on the World Health Organization referent (de Onis et al. 2009). Rapid growth was defined as a *z*-score change of > 0.67 between birth and 6 months of age, and slow growth was defined as a *z*-score change of < 0.67 (Monteiro and Victora 2005). Results were consistent with defining rapid growth using internal standardization of the sample or the U.S. Centers for Disease Control and Prevention referent (Kuczmarski et al. 2002) (data not shown). Additionally, to estimate effects of rapid early growth and OC exposure on later nutritional status, we estimated associations with BMI-for-age *z*-scores at 14 months of age. BMI *z*-scores ≥ 1.44, corresponding to the 85th percentile typically used to define child overweight, were defined as elevated. Consistent results were obtained classifying 14-month weight status using the U.S. referent and weight-for-length *z*-scores (data not shown).

### OC exposures

Maternal first-trimester serum levels of DDE, HCB, DDT [2,2-bis(*p*-chlorophenyl)-1,1,1-trichloroethane], β-hexachlorohexane (βHCH), and polychlorinated biphenyl (PCB) congeners 118, 138, 153, and 180 were measured by the Public Health Laboratory of the Basque government, using methods described elsewhere (Goñi et al. 2007). Because 99% of the sample had DDT levels below the limits of detection (LODs), this OC was excluded from the analysis. All subjects had detectable levels of DDE. For the remaining OCs, with up to approximately 10% below the LODs, levels were adjusted for lipids as prescribed by Phillips et al. (1989). Values below the LOD were imputed as LOD/2.

### Other variables

Maternal age, parity, education level, prepregnancy weight, country of origin, and occupation, along with paternal height, weight, and occupation, were reported by mothers at recruitment. Occupation data were used to assess social class (Instituto Nacional de Estadística 1994). Maternal height (nearest 0.5 cm) was used to calculate prepregnancy BMI (weight in kilograms/height in square meters) to classify women as normal weight, overweight (BMI ≥ 25 kg/m^2^), or obese (≥ 30 kg/m^2^). Weight gains throughout pregnancy from prenatal visit records were used to validate prepregnancy weight and to estimate gestational weight gain adequacy (Institute of Medicine 2009). Information on tobacco use throughout pregnancy was self-reported in the first and third trimesters, child sex was recorded at birth, and breast-feeding duration was reported in postnatal questionnaires. All analyses of rapid growth were conducted in the sample with complete data for the main exposure, outcome, and covariates (*n* = 518); analyses of BMI at 14 months had slightly fewer observations because of missing values for this variable (*n* = 502). Within the analysis sample with complete exposure and outcome data, there were few additional missing values for covariates (*n* = 17 and *n* = 16, respectively, for analyses of rapid growth and BMI at 14 months).

### Statistical analysis

Characteristics of rapid versus average/slow growers, including maternal OC levels and infant anthropometric measures, were described using means ± SD or proportions, with chi-square, analysis of variance, Student’s *t*-test, or Wilcoxon–Mann–Whitney tests applied as appropriate to assess statistical significance (*p* < 0.05 considered significant for main effects). Because maternal weight status was found to modify the effect of OC exposures on rapid growth (*p* < 0.10 used to define significance for interactions), OC levels were also described separately among normal-weight versus overweight women. Because distributions were skewed, geometric means were used to describe levels of OCs and significance assessed using Wilcoxon–Mann–Whitney tests on log-transformed data. Relationships among OCs were assessed using Spearman’s correlations and log-transformed data.

Generalized linear models (GLMs) for binary outcomes were used to estimate relative risks (RRs) and 95% confidence intervals (CIs) for associations between increasing levels of each OC and rapid early growth (Zou 2004). To assess the linearity of relationships, generalized additive models were used to examine the shape of the dose–response relationship adjusted for relevant confounders (Hastie and Tibshirani 1995). When relationships were nonlinear, OC quartiles were used; log-transformed continuous variables were used for linear relationships. GLMs were also used to assess how both rapid early growth and prenatal OCs—separately as well as adjusting simultaneously—related to the risk of elevated BMI at 14 months.

Maternal characteristics (age, education, social class, parity, prepregnancy weight status, gestational weight gain, smoking during pregnancy, weeks of gestation at blood sampling, triglycerides, cholesterol) and other potential influences on postnatal growth, including child’s sex, gestational age at birth and SGA status, paternal height and weight, and breast-feeding duration, were considered as potential confounders of associations between prenatal OCs and infant growth. Covariates retained in the final models included infants’ exact age at measurement and variables that resulted in a change in estimate ≥ 10%.

In supplementary analyses, we confirmed that excluding SGA (*n* = 28) or low-birth-weight (*n* = 15) infants, among whom catch-up growth may be normative, did not meaningfully change results. Results for individual PCB congeners, similar to those for the sum of PCBs, are not shown. Excluding women born outside Spain (*n* = 50), who may differ in terms of OC exposures and infant care practices, and excluding nonwhite (*n* = 13) or underweight (BMI < 18; *n* = 30) mothers at the start of pregnancy also had no meaningful influence on results (data not shown).We examined whether child sex modified effects of OCs on growth; interactions did not reach significance (*p* > 0.10). Finally, we confirmed that associations did not change when meaningfully restricting the referent to average growers (*n* = 276, 53.28% of the sample) rather than average/slow growers (data not shown). All analyses used STATA, version 10.1 (StataCorp LP, College Station, TX, USA).

## Results

Though rapid growers weighed less at birth (mean difference, 293 g), by 6 months of age these infants were significantly heavier than were average/slow growers (891 g) (Table 1). Rapid growers remained taller and heavier at 14 months.

### OCs and rapid growth among normal-weight versus overweight mothers

HCB and βHCH were highly correlated (Spearman’s *r* = 0.79, log-transformed variables), with more moderate correlations with DDE (HCB–DDE, *r* = 0.29; βHCH–DDE, *r* = 0.39; PCBs–DDE, *r* = 0.39) and PCBs (PCBs–HCB, *r* = 0.51; PCBs–βHCH, *r* = 0.40). Overall, mean levels of all OCs were similar among rapid and average/slow growers (Table 2). However, among infants of women of normal prepregnancy weight, mean levels of DDE, HCB, and βHCH were significantly higher among rapid growers than among average/slow growers (*p* < 0.05). Neither the sum of PCBs nor individual PCBs (data not shown) were associated with rapid growth. In contrast, among infants of women overweight before pregnancy, mean OC levels were lower among rapid growers than among average/slow growers, although differences were not statistically significant.

Among infants whose mothers were of normal prepregnancy weight, associations between increasing prenatal DDE exposure and rapid growth were nonlinear, with an increasing predicted risk followed by an apparent decline in the top 3% of exposure [> 750 ng/g; ~ 6.6 ng/g on the log scale; see Supplemental Material, Figure 1 (doi:10.1289/ehp.1002169)]. Therefore, OC exposures were categorized as quartiles to examine dose–response relationships between OCs and rapid growth (Table 3). Except for PCBs (data not shown), which were essentially unrelated to rapid growth (21.3%/25.2% rapid growers in the top/bottom quartile), risk of rapid growth among infants of normal-weight mothers rose with increasing quartiles of exposure to each OC. Disparities in rapid growth in this group were greatest for DDE, with a nearly 3 times greater frequency (29.8% vs. 11.7%; *p* < 0.05) in the highest than in the lowest quartiles. After multivariable adjustment, exposures above the lowest quartile of DDE were associated with a 2.4 times greater risk of rapid growth among normal-weight mothers (*p* < 0.05). Associations were similar and remained significant after adjusting for other OCs. For HCB and βHCH, however, associations with rapid growth were weakened and not significant after adjusting for DDE (Table 3). Because HCB and βHCH were highly correlated, we examined results adjusting for either OC but not both simultaneously; findings were very similar (data not shown).

In the group of infants with normal-weight mothers, risk of rapid growth associated with the top quartile of DDE exposure was slightly strengthened by excluding the subjects (*n* = 15) with DDE > 750 ng/g (6.6 ng/g, log scale), none of whom were rapid growers (RR, 2.76; 95% CI, 1.36–5.62). Alternatively, excluding foreign-born mothers (73.3% of subjects at this level of exposure) had little effect (fourth vs. first quartile: RR = 2.35; 95% CI, 1.15–5.01). Results were also consistent after excluding *n* = 28 SGA infants (RR = 2.65; 95% CI, 1.26–5.58), or *n* = 15 low-birth-weight infants (RR = 3.04; 95% CI, 1.45–6.35). Using log DDE as a continuous measure, the RR per one-unit increase (2.72 ng/g on the original scale) was 1.24 (95% CI, 1.02–1.50) for all subjects and was 1.60 (95% CI, 1.22–2.09) after excluding the top 3% of exposure. Compared with infants of normal-weight mothers, infants in the smaller sample with overweight mothers showed associations that were inverse but not significant.

To maximize cell sizes in models stratified by maternal overweight, we used tertiles to explore whether associations between OCs and rapid growth varied by infant sex. Although DDE–child sex interaction terms did not achieve statistical significance (*p* = 0.17 overall; *p* = 0.14 among normal-weight mothers), DDE was more strongly associated with rapid growth in boys than in girls. Among boys with normal-weight mothers, the risk of rapid growth in the intermediate and highest tertiles of DDE was more than three times higher than in the lowest tertile (highest vs. lowest tertile: multivariable-adjusted RR = 3.61; 95% CI, 1.36–9.56; *n* = 6 rapid growers in the referent group). The intermediate (RR = 1.71; 95% CI, 0.90–3.23) but not the highest (RR = 0.90; 95% CI, 0.40–2.08) tertile of DDE was positively, although not significantly, associated with rapid growth in girls (*n* = 13 rapid growers in the referent group).

### OCs, rapid growth, and BMI at 14 months

At 14 months, 13.9% of infants had elevated BMI *z*-scores. Rapid growers were more likely than were average/slow growers to have an elevated BMI *z*-score (35.5% vs. 7.1%; *p* < 0.05). After multivariable adjustment, rapid growth in the first 6 months was associated with a 5 times higher risk of elevated 14-month BMI, with similar associations among normal-weight and overweight mothers. In addition, prenatal DDE, but not other OCs, was associated with elevated BMI *z*-scores at 14 months among infants of normal-weight mothers (Table 4).

## Discussion

There is growing awareness that factors aside from increasing intakes of energy-dense foods and declining physical activity may play key roles in the obesity epidemic (McAllister et al. 2009). It is also increasingly apparent that long-term obesity risk may be evident from rapid growth initiated in the first months of life (Ong and Loos 2006). In this study, we found that among mothers not overweight or obese before pregnancy, higher DDE levels in first-trimester serum were strongly associated with two indicators of early obesity risk in their children: rapid weight gain in the first 6 months of life and elevated BMI *z*-scores at 14 months. These results are consistent with two earlier studies that reported associations between prenatal DDE exposure and obesity later in life (Karmaus et al. 2009; Verhulst et al. 2009). However, this analysis suggests, to our knowledge for the first time, that fetal DDE exposure may promote rapid growth starting in the immediate postnatal period, consistent with the hypothesis that early-life exposure to EDCs may promote obesogenic changes in metabolism (Heindel and vom Saal 2009).

Regardless of DDE exposure levels and maternal prepregnancy weight status, children with rapid growth in the first months of life had a 5 times higher risk of attaining BMIs exceeding the 85th percentile by 14 months of age. Although changes in feeding and other care practices could influence future growth trajectories in these children (Karaolis-Danckert et al. 2006), numerous studies suggest heavier weight status at 1–2 years is predictive of later obesity (Baird et al. 2005).

The “environmental obesogen” hypothesis postulates that the developmental period is a vulnerable window during which transient environmental exposures may alter the regulation of weight or adiposity over the long term, perhaps as a consequence of epigenetic changes influencing gene expression or protein regulation (Heindel and vom Saal 2009; Lee et al. 2009). *In vitro* and animal experiments suggest that some EDCs may upregulate adipogenesis or promote fat storage through mechanisms such as the activation of retinoid-X receptor, peroxisome proliferator activated receptor-γ, or glucocorticoid receptor pathways or by disruption of estrogen receptor signaling (Grün and Blumberg 2009; Sargis et al. 2010). The issue is of concern because exposure to many EDCs is widespread, as a result of long half-lives and extensive use in agriculture and industry (Lang et al. 2008; Wilson et al. 2003). Despite evidence of obesogenicity for a few chemicals in animal studies, however, few empirical data have linked such exposures to obesity in humans. In cross-sectional studies, higher levels of EDCs such as BPA and phthalates have been reported in obese than in normal-weight subjects, although because these EDCs are fat soluble, it is not clear whether this partly reflects larger adipose tissue depots in fatter subjects (Lang et al. 2008; Stahlhut et al. 2007).

DDE, a metabolite of the pesticide DDT, has not been explored in animal studies on environmental obesogens. However, experimental data indicate that DDE does modulate endocrine function and influences gene expression after binding to nuclear receptors (Li et al. 2008; Sonnenschein and Soto 1998). Although DDT use is prohibited in most industrialized countries, including Spain, high concentrations of DDE continue to be reported in human biomarker studies because of its long half-life and persistence in water, sediments, and the food chain. DDE levels in this sample were similar to those reported both among pregnant women by Verhulst et al. (2009) and in first-trimester samples in the U.S. National Health and Nutrition Examination Survey (Wang et al. 2009).

Other than DDE, the OCs examined in this study were not independently associated with either rapid early weight gain or BMI at 14 months. For PCBs, the absence of association is consistent with data from Karmaus et al. (2009), although Verhulst et al. (2009) reported a very small increase in BMI *z*-scores associated with PCB exposure (0.003 for each 1-ng/g increase). Verhulst et al. (2009) reported HCB exposure in a range similar to those in our sample to be unassociated with BMI through 3 years of age. Prenatal HCB levels here were three times lower than in a previous study (Smink et al. 2008) reporting an association with later childhood obesity (median, 0.25 vs. 0.68 ng/mL). Further research is needed to ascertain whether disparities in exposure levels or different mechanisms of action for either individual OCs or mixtures might explain why we observed significant positive associations only for DDE.

Excluding SGA or low-birth-weight infants had little influence; indeed, results were slightly strengthened after excluding either group. This is important given that rapid early growth was initially thought to increase obesity risk predominantly in SGA infants, who might be programmed *in utero* to use dietary energy efficiently, perhaps increasing susceptibility to excess weight gain when exposed to energy-dense foods and sedentary environments (Dunger and Ong 2005). It is now evident, however, that rapid early weight gain is linked with obesity among infants of average size (Karaolis-Danckert et al. 2006; Leunissen et al. 2009).

None of the small number of infants at the very highest levels of maternal DDE exposure (> 750 ng/g), primarily of foreign-born mothers, experienced rapid growth in the first 6 months. We cannot determine whether this occurred by chance, or whether very highly exposed children might be at reduced risk of rapid weight gain. However, similar opposing patterns of risk at low versus high exposure levels have been reported in other studies on health effects of xenoestrogens (Wadia et al. 2007; Weltje et al. 2005). Reasons for the differential associations between fetal DDE exposure and rapid growth by maternal prepregnancy weight status are also uncertain; although the sample was small, associations between DDE and early rapid growth appeared to be negative among overweight mothers. In our study, children of obese mothers were at increased risk of overweight for reasons independent of chemical exposures: these infants had higher weight and BMI *z*-scores at birth and at 6 and 14 months of age regardless of prenatal DDE levels (data not shown). Experimental evidence suggests that offspring of obese mothers may be at increased risk of obesity, possibly due to genetic susceptibility or perhaps to epigenetic changes (Waterland et al. 2008; Wu and Suzuki 2006). For example, lower intakes of nutrients such as folate, which has been reported among obese pregnant women (Rifas-Shiman et al. 2009), may increase risk of offspring obesity, given animal studies showing dietary supplementation with methyl donors including folate to be associated with reduced body weight in subsequent generations (Waterland et al. 2008). Animal studies have also reported stronger obesogenic effects of high-fat diets in offspring of obese than of normal-weight mothers (White et al. 2009). Moreover, observational studies have reported breast-feeding to be more strongly protective against child obesity among overweight mothers than among normal-weight mothers (Li et al. 2005). Very early growth patterns among infants of overweight and obese mothers may, therefore, be more strongly related to factors other than OC exposure.

Although interactions with child sex did not reach significance either overall or after stratifying by maternal weight status (*p* > 0.10), associations between DDE and rapid growth were stronger in boys. Some studies have suggested that effects of periconceptional exposures to EDCs and other factors may be sex-specific (Gabory et al. 2009; Tobi et al. 2009). The modest sample size may have lacked power to detect statistically significant interactions.

Although prenatal DDE exposure was associated with rapid weight gain and BMI, no direct measures of adiposity were available. However, previous studies have reported that rapid weight gain was more strongly associated with direct measures of body fatness than with BMI (Holzhauer et al. 2009; Karaolis-Danckert et al. 2006). We measured prenatal DDE only in maternal serum collected in early pregnancy; levels have been observed to increase later in pregnancy because of metabolic changes during gestation (Torres-Sánchez et al. 2007; Wang et al. 2009). However, studies using repeated measures from multiple trimesters have found that only first-trimester DDE was associated with early neurodevelopment and genital structure alterations (Torres-Sánchez et al. 2007, 2008). Although potential mechanisms are uncertain, associations with first-trimester measures are consistent with hypotheses involving epigenetic changes, because such effects have been observed to be more frequent for periconceptional than for later exposures (Lee et al. 2009; Tobi et al. 2009). Other limitations include the modest sample size, resulting in few rapid growers in the referent group and fairly wide conference intervals. An important strength is the large number of potential confounders considered, including birth weight and weight gain in pregnancy, which were only weakly (Spearman’s *r* < 0.10) correlated with prenatal DDE and did not confound associations. Although follow-up thus far is short, future analyses will assess longer-term effects of OCs on postnatal growth.

## Conclusion

We found that among normal-weight mothers, prenatal DDE exposure was associated with an increased risk of rapid growth in the first 6 months of life, as well as elevated BMI at 14 months. Other OCs examined were not associated with increased risk of rapid growth. Given the large body of evidence indicating that rapid weight gain in the first months of life increases long-term risk of obesity and metabolic disorders, these data provide support for a possible role of prenatal exposure to DDE in programming these health problems. Future research is needed to assess whether the association may be causal and what the underlying mechanisms might be.

## Figures and Tables

**Table 1:** Characteristics of rapid, average, and slow growers.

Characteristic	Average/slow growers (*n* = 393)	Rapid growers (*n* = 125)	*p*-Value^a^
Male sex	201 (51.2)	64 (51.2%)	0.991

Birth characteristics			

Birth weight (kg)	3.34 ± 0.02	3.04 ± 0.04	< 0.001
Birth weight *z*-score	0.069 ± 0.039	−0.574 ± 0.076	< 0.001
Birth length (cm)	49.69 ± 0.09	48.98 ± 0.16	< 0.001
Gestational age (weeks)	39.90 ± 0.06	39.53 ± 0.10	0.003
SGA	17 (4.34)	21 (16.80)	< 0.001
Low birth weight (< 2,500 g)	3 (0.76)	12 (9.60)	< 0.001

Infant growth at 6 and 14 months			

Weight at 6 months (kg)	7.42 ± 0.04	8.29 ± 0.08	< 0.001
Weight *z*-score at 6 months	−0.28 ± 0.80	0.68 ± 0.83	< 0.001
Weight at 14 months (kg)	10.07 ± 0.05	11.12 ± 0.12	< 0.001
Length at 14 months (cm)	77.37 ± 0.15	78.75 ± 0.24	< 0.001
BMI at 14 months (kg/m^2^)	16.64 ± 1.33	18.26 ± 1.51	< 0.001
Weight *z*-score (14 months)	0.12 ± 0.85	0.96 ± 0.99	< 0.001
Length *z*-score (14 months)	−0.20 ± 1.04	0.33 ± 0.96	< 0.001
BMI *z*-score (14 months)	0.34 ± 0.82	1.06 ± 0.95	< 0.001

Parental characteristics			

Maternal age (years)	31.78 ± 0.21	31.63 ± 0.41	0.983
Maternal height (m)	1.62 ± 0.00	1.63 ± 0.01	0.592
Maternal parity (firstborn)	216 (54.96)	76 (60.80)	0.252
Maternal overweight/obesity	112 (28.5)	32 (25.6)	0.529
Mother born outside Spain	38 (9.74)	12 (9.76)	0.997
Maternal education			
Primary or less	94 (23.98)	42 (33.87)	
Secondary	166 (42.35)	48 (38.71)	
University	132 (33.67)	34 (27.42)	0.084
Manual occupation, mother^b^	181 (46.1)	66 (52.8)	0.189
Paternal height (m)	1.76 ± 0.004	1.77 ± 0.007	0.257
Paternal overweight/obesity	209 (53.7)	71 (57.7)	0.438
Manual occupation, father	206 (56.0)	73 (61.3)	0.304

Values are mean ± SD or *n* (%). Data are for the complete analysis data set; missing values for individual covariates ranged from 2 to 14 observations, with *n* = 17 observations lost due to missing values for all covariates combined. There was no significant difference in the proportion missing among average/slow versus rapid growers (*p*-values, two-sample test for proportions > 0.3). World Health Organization data (de Onis et al. 2009) were used to derive *z*-scores.

a *t*-Test (for normally distributed) or Wilcoxon–Mann–Whitney for continuous variables; chi-square test for categorical variables.

b Manual occupation: skilled or partially skilled manual workers during pregnancy or most recent job before pregnancy.

**Table 2:** Lipid-adjusted OC levels in first-trimester maternal serum for rapid and average/slow growers [geometric mean (95% (CI)].

OC	Percent < LOD^a^	Average/slow growers	Rapid growers
All infants, ng/g lipid (*n* = 518)			

DDE	0.0	125.40 (115.03–136.70)	135.59 (119.48–153.87)
HCB	8.5	23.38 (19.00–28.78)	26.53 (18.47–38.13)
βHCH	10.0	17.79 (14.37–22.04)	20.46 (13.98–29.94)
∑PCBs	7.7	43.72 (36.36–52.56)	47.99 (34.84–66.11)

Normal-weight mothers, ng/g lipid (*n* = 374)			

DDE*	0.0	120.76 (108.70–134.15)	146.78 (127.97–168.35)
HCB*	9.1	18.82 (14.51–24.42)	31.21 (21.89–44.50)
βHCH*	10.4	15.09 (11.57–19.70)	25.54 (17.55–37.19)
∑PCBs	7.2	45.20 (36.02–56.73)	66.96 (51.20–87.58)

Overweight mothers, ng/g lipid (*n* = 144)			

DDE	0.0	137.84 (118.71–160.07)	107.68 (80.46–144.10)
HCB	6.9	40.28 (29.64–54.74)	16.56 (6.11–44.89)
βHCH	9.0	26.89 (19.20–37.66)	10.73 (3.85–29.86)
∑PCBs	9.0	40.21 (29.50–54.80)	18.22 (7.14–46.51)

∑ PCBs refers to sum of congeners 118, 138, 153, and 180. Among normal-weight mothers, *n* = 93 rapid growers (24.9%); among overweight mothers, *n* = 32 rapid growers (22.2%). All subjects had detectable levels of DDE.

a LOD = 0.071 ng/mL serum for all OCs; values below this level set to LOD/2.

* Wilcoxon–Mann–Whitney *p*-value < 0.05.

**Table 3:** Maternal OC levels and risk of rapid infant weight gain: number of rapid growers in each exposure group.

OC quartile	Normal-prepregnancy-weight mothers (*n* = 374)			Overweight/obese mothers (*n* = 144)		
	Multivariable adjusted		Plus all OCs^a^ [RR (95% CI)]	Multivariable adjusted		Plus all OCs^a^ [RR (95% CI)]
	*n* (%)	RR (95% CI)		*n* (%)	RR (95% CI)	
DDE (ng/g lipid)						

≤ 71.71	11 (11.7)	1.00	1.00	14 (38.9)	1.00	1.00
71.71–116.92	27 (28.7)	2.42 (1.25–4.67)	2.21 (1.14–4.29)	5 (14.3)	0.48 (0.19–1.23)	0.61 (0.22–1.69)
116.92–186.17	27 (29.3)	2.47 (1.24–4.92)	2.21 (1.07–4.54)	8 (21.1)	0.66 (0.29–1.54)	0.87 (0.41–1.87)
> 186.17	28 (29.8)	2.47 (1.22–5.00)	2.16 (1.05–4.45)	5 (14.3)	0.43 (0.18–1.06)	0.43 (0.15–1.24)

HCB (ng/g lipid)						

≤ 22.84	20 (18.0)	1.00	1.00	8 (42.1)	1.00	1.00
22.84–41.00	20 (21.7)	1.17 (0.66–2.08)	0.92 (0.50–1.69)	10 (27.0)	0.46 (0.21–0.99)	0.17 (0.07–0.45)
41.00–66.28	26 (28.0)	1.54 (0.90–2.64)	1.14 (0.60–2.17)	5 (13.5)	0.21 (0.08–0.52)	0.10 (0.03–0.35)
> 66.28	27 (34.6)	1.78 (0.96–3.32)	1.20 (0.56–2.56)	9 (17.6)	0.19 (0.07–0.57)	0.11 (0.03–0.51)

βHCH (ng/g lipid)						

≤ 21.70	16 (15.8)	1.00	1.00	8 (27.6)	1.00	1.00
21.70–32.23	25 (25.0)	1.67 (0.95–2.93)	1.45 (0.81–2.62)	10 (34.5)	1.19 (0.48–2.96)	3.88 (1.40–10.70)
32.23–47.28	22 (24.7)	1.53 (0.83–2.85)	1.13 (0.54–2.36)	6 (14.6)	0.44 (0.16–1.18)	1.95 (0.60–6.36)
> 47.28	30 (35.7)	2.08 (1.11–3.87)	1.42 (0.67–3.01)	8 (17.8)	0.52 (0.16–1.67)	2.02 (0.63–6.48)

Multivariable model adjusted for exact age at examination, gestational age, any breast-feeding at 6 months, firstborn child, maternal smoking during pregnancy, education, and maternal age at delivery.

a Additionally adjusted for quartiles of the other two OCs shown.

**Table 4:** Associations for rapid early growth, maternal OC levels, and elevated BMI *z*-score (≥ 1.44) at 14 months [RR (95% CI)].

Variable	All mothers (*n* = 502)	Normal-prepregnancy-weight mothers (*n* = 365)	Overweight/obese mothers (*n* = 137)	Interaction *p*-value (× maternal weight status)
Multivariable models				

DDE (per log ng/g lipid)	1.22 (0.96–1.55)	1.64 (1.24–2.18)	0.93 (0.60–1.45)	0.086
HCB (per log ng/g lipid)	0.97 (0.86–1.10)	1.02 (0.88–1.18)	0.74 (0.63–0.87)	0.680
βHCH (per log ng/g lipid)	1.03 (0.93–1.15)	1.06 (0.90–1.23)	0.97 (0.84–1.12)	0.678
Rapid growth in the first 6 months	4.71 (3.02–7.35)	5.18 (2.92–9.18)	5.02 (2.56–9.85)	0.420

Mutually adjusted contaminants				

DDE (per log ng/g lipid)	1.40 (1.12–1.75)	1.50 (1.11–2.03)	1.22 (0.81–1.84)	0.270
HCB (per log ng/g lipid)	1.00 (0.85–1.17)	0.99 (0.85–1.15)	0.91 (0.74–1.12)	0.340
βHCH (per log ng/g lipid)	0.96 (0.81–1.12)	0.92 (0.78–1.09)	0.95 (0.76–1.18)	0.601
Rapid growth in the first 6 months	4.85 (3.12–7.54)	5.41 (2.88–10.15)	4.40 (2.09–9.28)	0.592

Data are multivariable-adjusted RRs (95% CIs) for likelihood of having an elevated BMI *z*-score at 14 months. All models adjusted for exact age at examination, maternal country of birth, maternal age at delivery, smoking during pregnancy, and parental overweight; rapid growth was included in all models along with either individual or multiple contaminants, as indicated.
